# Health information technology capacity at federally qualified health centers: a mechanism for improving quality of care

**DOI:** 10.1186/1472-6963-13-35

**Published:** 2013-01-31

**Authors:** Jemima A Frimpong, Bradford E Jackson, LaShonda M Stewart, Karan P Singh, Patrick A Rivers, Sejong Bae

**Affiliations:** 1 Department of Health Policy and Management, Mailman School of Public Health, Columbia University, New York, NY, 10032, USA; 2 Division of Preventive Medicine, University of Alabama at Birmingham, 1717 11th Ave. South - Medical Towers Bldg, Birmingham, AL, 35294-4410, USA; 3 Public Administration Program, Department of Political Science, Southern Illinois University, 1000 Faner Drive, Carbondale, IL, 62901, USA; 4 College of Applied Science & Arts, Southern Illinois University, 1365 Douglas Drive, Carbondale, IL, 62901, USA

**Keywords:** Health information technology, Federally qualified health centers, Electronic medical records, Quality of care, Patient reminder/notification, Adoption of technology, Meaningful use, Care coordination

## Abstract

**Background:**

The adoption of health information technology has been recommended as a viable mechanism for improving quality of care and patient health outcomes. However, the capacity of health information technology (i.e., availability and use of multiple and advanced functionalities), particularly in federally qualified health centers (FQHCs) on improving quality of care is not well understood. We examined associations between health information technology (HIT) capacity at FQHCs and quality of care, measured by the receipt of discharge summary, frequency of patients receiving reminders/notifications for preventive care/follow-up care, and timely appointment for specialty care.

**Methods:**

The analyses used 2009 data from the National Survey of Federally Qualified Health Centers. The study included 776 of the FQHCs that participated in the survey. We examined the extent of HIT use and tested the hypothesis that level of HIT capacity is associated with quality of care. Multivariable logistic regressions, reporting unadjusted and adjusted odds ratios, were used to examine whether ‘FQHCs’ HIT capacity’ is associated with the outcome measures.

**Results:**

The results showed a positive association between health information technology capacity and quality of care. FQHCs with higher HIT capacity were significantly more likely to have improved quality of care, measured by the receipt of discharge summaries (OR=1.43; CI=1.01, 2.40), the use of a patient notification system for preventive and follow-up care (OR=1.74; CI=1.23, 2.45), and timely appointment for specialty care (OR=1.77; CI=1.24, 2.53).

**Conclusions:**

Our findings highlight the promise of HIT in improving quality of care, particularly for vulnerable populations who seek care at FQHCs. The results also show that FQHCs may not be maximizing the benefits of HIT. Efforts to implement HIT must include strategies that facilitate the implementation of comprehensive and advanced functionalities, as well as promote meaningful use of these systems. Further examination of the role of health information systems in clinical decision-making and improvements in patient outcomes are needed to better understand the benefits of HIT in improving overall quality of care.

## Background

The Health Information Technology for Economic and Clinical Health (HITECH) Act and the American Recovery and Reinvestment Act (ARRA), enacted in 2009, promote the adoption of health information technology by hospitals (and other health delivery care organizations) and care providers [1,2]. Health information technology (HIT) is defined as “a variety of electronic methods used to manage information about people’s health and health care, on both the individual and group level” [3]. The goal of these federal policies is to promote the use of HIT-based information in a way that improves care delivery and health outcomes (i.e., meaningful use) [2,4]. Meaningful use of HIT includes improving coordination and quality of care and engaging patients and families in the care delivery process [5-7]. Potential benefits include the improvements of individual health as well as enhancements in the performance of health service providers [8]. Overall, HIT is expected to improve quality of care, reduce costs, and facilitate patient-centeredness by using technological advances to engage patients as active participants in the care delivery process [7,9].

Numerous studies have examined the effects of HIT on service delivery and quality of care and suggest that there are improvements in cost savings and patient outcomes [10-13]. The adoption and use of HIT has also been associated with improvements in process measures (i.e., provision of preventive service, prescribing therapies), [6] quality improvement [14,15] and reductions in medication errors [12]. Other studies show that the use of electronic health records as a tool for improving clinical workflow and process redesign are positively associated with quality improvements [4,16]. Amarasingham et al. examined whether greater automation of information was associated with reductions in inpatient mortality, length of stay, costs, and complications, and found that hospitals which implemented health technology (including electronic notes and records, order entry, and clinical decision-support) had better outcomes as indicated by fewer complications and lower mortality rates and costs [13]. A review of HIT on quality, efficiency, and cost of medical care reported that the implementation of multifunctional HIT systems was associated with quality and efficiency benefits [17]. A more recent systematic review by Buntin et al. also reported that HIT has an overwhelmingly positive effect on quality of care and care efficiency. The authors noted the broad reach of improvements associated with the adoption and use of HIT: access to care, patient safety, preventive services, and patient satisfaction [2].

Although the diffusion of electronic health records in the health care industry had been slow, [18] there has been a rapid increase in HIT adoption following the AARA. The strong emphasis in the ARRA on adoption and meaningful use of HIT, supported by the creation of incentives for meaningful use, has had a great effect on the rapid increase in the acquisition and utilization of HIT in various health care settings [2,13]. However, inequality exists with regard to availability of information technology across facility types. Specifically, there is sparse knowledge about the use and functionalities of health information technology in federally qualified health centers (FQHCs) and the relationship between quality improvements and the use of health information technology in FQHCs. FQHCs are community health centers that provide primary care services to vulnerable and underserved populations in rural and urban areas. One study reported that health centers serving the most vulnerable patients were less likely to have HIT systems, and among those with these systems, only 50% had the minimum set of functionalities [19].

In order for FQHCs’ to absorb the expected increase in demand and improve quality of care, they must leverage HIT as a tool for improving service delivery and patient outcomes [2,20]. Given the potential benefits of HIT, there is a need to examine the availability of HIT at health care organizations that predominantly serve underrepresented populations, as well as associations between HIT capacity and performance. Particularly, the essential role of health centers in providing health care to millions of Americans [21] underscore the need for technologies and strategies that improve access to high quality care [22]. Therefore, this study examined the health information technology capacity of FQHCs to determine associations with improved quality of care. The goals of the study are twofold: 1) identify factors associated with higher HIT capacity at FQHCs, and 2) examine associations between HIT capacity and quality of care in federally qualified health centers. We define HIT capacity by the functionalities of the health information system available in FQHCs. The measure of HIT capacity used in the paper is relevant in that it is informed by functionalities required for meaningful use. Specifically, the measure was operationalized based on correspondence with the HITECH meaningful use objectives [23]. Meaningful use of HIT is expected to improve quality, safety, and the effectiveness of patient-centered care. Specifically, the measures for this study include use of electronic health records for exchange of information on quality of care, electronic prescriptions, and clinical decision-support [23] Lastly, the outcome measures examined in this study are important in that they have been significantly associated with increased adherence to treatment, utilization of health services, receipt of preventive services, and treatment outcomes [24-26].

## Methods

### Data

Data for this study come from the 2009 Commonwealth Fund National Survey of Federally Qualified Health Centers and was used with permission from the Commonwealth Fund [7]. The survey collects information on organizational level measures, including access to care, coordination of care across settings, engagement in quality improvement and reporting, HIT adoption, and the ability to serve as patient-centered medical homes. The survey was conducted among executive directors or clinical directors at FQHCs. The sample was selected from a list of all FQHC grantees that have at least one site that is a community-based primary care clinic. Of the 1,007 FQHCs that were sent the questionnaire, 795 responded, for a response rate of 79%, and 776 were included in the analysis. Sampling weights were generated for the data based on number of patients, number of sites, region, and urbanicity in order to more precisely reflect the universe of community health centers [7].

### Outcome measures

The outcome measures of interest are quality of care, measured by receipt of discharge summary, frequency of patients receiving reminders/notifications for preventive care/follow-up care, and timely appointment for specialty care. Receipt of discharge summaries and patient reminder notifications for preventive or follow-up care were measured using the following questions: “Your center receives a discharge summary or report from the hospital to which your patients are usually admitted” and “patients are sent reminder notices when it is time for regular preventive or follow-up care (e.g., flu vaccine or HbA1C for diabetic patients”, respectively. The available answer categories for the questions were “usually (75–100% of the time); often (50–74% of the time); sometimes (25–49% of the time); rarely (1–24% of the time); or never.” Timely appointment was measured by how difficult it was for providers to “obtain timely appointments for office visits with specialists or subspecialists outside your center for uninsured patients? Response categories for the question were “easy, somewhat difficult, and very difficult.”

The outcome variables for measuring receipt of discharge summary and patient reminder notifications for prevention or follow-up care originally had multiple levels. Frequency distributions of these variables showed that there were small cell frequencies for several categories. Therefore, measures were collapsed into two different categories. Receipt of discharge summary and patient reminder notifications were dichotomized to “usual/often” or “some/rarely/never.” Obtaining timely appointment for specialty care was dichotomized to “easy/somewhat difficult or very difficult.”

### Primary explanatory variable

The survey asked whether health facilities use electronic health records throughout the health center (yes/no), and whether their largest site had a computerized system for 15 other functionalities. Survey items supporting HIT capacity fall under three categories: routine use of technology for notes, medications, test, and clinical prompts; computerized process for patient registries; and computerized process for tracking test and reminder/alerts. Health Information Technology (HIT) capacity was scored based on a health centers’ possession of the 16 functionalities of technologies (Table 1).

**Table 1:** Survey items Supporting Health Information Technology (HIT) capacity in federally qualified health centers

**Category (number of items)**	**Survey items**
**Routine use of technology for notes, medications, test, and clinical prompts (7)**	Use of electronic health records
	Electronic entry of clinical notes, including medical history and follow-up notes*
	Electronic ordering of laboratory tests
	Electronic access to patients laboratory test result*
	Electronic prescribing of medication*
	Electronic list of all medications taken by a patient (including those prescribed by other doctors)
	Electronic alerts or prompts about a potential problem with drug dose or drug interaction
**Computerized process for patient registries (6)**	List of patients by diagnosis (e.g., diabetes or hypertension)*
	List of patients by health risk (e.g., smokers)*
	List of patients by lab result (e.g., HbA1C>9.0)*
	List of patients who are due/overdue for tests/preventive care (e.g., flu vaccine due)
	List of patients taking a specific medication (e.g., patients on ACE inhibitors)
	List of panel of patients by provider*
**Computerized process for tracking test and reminder/alerts (3)**	Does the provider receive an alert/prompt at point of care for appropriate care services needed by patients (e.g., pap smear or immunizations due)
	Does the provider receive an alert or prompt to provide patients with test results
	Are laboratory tests ordered, tracked until results reach clinicians

Note. Asterisk (*) refers to "minimum required" or “must-haves” functionalities of a health information technology (HIT) *system.*

Due to the variation in the responses of centers to the 16 survey items, we classified FQHCs as either ‘Low’, ‘Medium’, or ‘High’ HIT capacity in order to examine differences from the observed levels. Those FQHCs that had fewer than four of seven “minimum required” or “must-have” functionalities for a HIT system (see Table 1) were classified as ‘Low’. Health centers that had four of the seven “minimum required” and at least one to six functionalities from the remaining items (for a total between 5 and 10 items from the full list) were categorized as ‘Medium’. Finally, health centers that had at least four "minimum-required" functionalities and a total between 10 and 16 functionalities were considered as having a ‘High’ HIT capacity. The “Low” category was based exclusively on the minimum set of functionalities. All “minimum requirement” items were nested within the full list of items. That is, the “medium” and “high” categories were measured using the full list of 16 functionalities. The main difference between the “minimum required” functionalities and the other items is the lack of certain decision-support functionalities for providers (i.e., prompts) and or tracking of tests.

### Statistical analysis

Frequencies and weighted percentages were generated for the distribution of characteristics of FQHCs. The finite population correction and sampling weight were incorporated into the analysis. Chi square tests were used to test the hypothesis that HIT capacity is associated with quality of care; and to examine the homogeneity between levels of each factor. With the three ordered categories of increasing HIT capacity (i.e., low, medium, high), ordinal Logistic regression was performed and the proportional odds assumptions were verified for all models.

In the analysis, we are interested in making comparisons of predicted outcomes after controlling for covariate distributions. We therefore used multivariable logistic regressions, reporting unadjusted and adjusted odds ratios, to examine whether FQHCs HIT capacity is associated with the outcome measures.

In the adjusted analyses we controlled for quality improvement initiatives (participation in HRSA health disparities collaborative project, overall measure of support for QI); workforce (physician vacancies, nurse vacancies); and usual source of care. The other control variables were incentives for patient satisfaction, hospital affiliation, size of facility, urbanicity, percent Medicaid patients, percent minority patients, and region. Size was categorized based on the total number of sites in each health center: small (1–3), medium (4–9), and large (10+). Urbanicity classified health centers as either urban (city, suburban and small town) or rural (rural and frontier). Region corresponds to the geographic location of the health center (Northeast, Midwest, South, and West). In different regions there is variability between how FQHCs may communicate/organize with other hospitals/communities in the area. In order to control for these differences we adjusted for Region and Urbanicity. Controlling for area-wide variability in surveys with complex design is a common approach [27,28]. Hospital affiliation was categorized based on health center affiliation with local hospitals (all affiliations, some affiliations, and none). All analyses were performed with SAS v9.3 using SURVEY procedures to account for the study design (SAS v9.3, SAS Institute Inc., Cary, NC, USA). Results were considered statistically significant if the p-values were less than 0.05.

## Results

### Characteristics of federally qualified health centers

The characteristics of the health centers can be found in Table 2. Of all the FQHCS, only 43% use electronic medical records, approximately 50 percent of FQHCs are classified as having Low HIT capacity, with 32% classified as having high HIT capacity. With regard to the outcome variables, approximately 57% of the sample stated that they usually or often receive a discharge summary from the hospital to which the patients were admitted. Only 35% of health centers reported usually or often sending patient reminders for preventive or follow-up care. About 67% reported that timely appointments for specialty care were very difficult. Almost all FQHCs have HRSA Disparity coordination (87%), with most health centers (52%) having fifty percent or more of their patients belonging to a minority group. There was a vacancy of physicians in 58% of the centers, and a vacancy of nurses in 42%. In terms of patient satisfaction, only 21% of the sample receives an incentive. Roughly half of all health centers had hospital affiliations.

**Table 2:** Characteristics of federally qualified health centers (N=776)

		**n**	**weighted %**	**p-value**
Use of EMR	Yes	321	42.71	***
	No	428	57.29	
HIT Capacity	Low	386	46.65	***
	Medium	146	21.73	
	High	239	31.62	
Receipt of discharge summaries	Usually (75–100% of the time)	276	36.15	***
	Often (50–74% of the time)	163	21.15	
	Sometimes (25–49 % of the time)	154	20.08	
	Rarely (1–24% of the time)	118	15.65	
	Never	50	6.97	
Patient Reminders	Usually (75–100% of the time)	140	18.44	***
	Often (50–74% of the time)	127	16.46	
	Sometimes (25–49% of the time)	182	23.65	
	Rarely (1–24% of the time)	185	24.81	
	Never	123	16.64	
Getting timely appointment - specialty care	Easy	49	6.44	***
	Somewhat Difficult	200	26.15	
	Very difficult	516	67.41	
Incentive for patient satisfaction	Yes	164	20.9	***
	No	598	79.1	
HRSA Disparity Coordination	Yes	633	87.32	***
	No	86	12.68	
Overall Measure of QI Support	Yes	453	57.84	***
	Some or none	319	42.16	
Physician vacancies	Yes	396	57.04	***
	No	292	42.96	
Nurse vacancies	Yes	270	42.93	***
	No	354	57.07	
Hospital Affiliation	All	377	47.81	0.2313
	Some or none	392	52.19	
Usual Source of Care	Usual/Often	689	89.84	***
	Some/Rarely/None	78	10.16	
Percent minority	Low (<5%)	116	15.62	***
	Medium (5–49%)	242	31.99	
	High (50% or more)	404	52.38	
Size	Large	107	15.8	***
	Medium	279	36.61	
	Small	379	47.59	
Region	Midwest	157	20.07	***
	South	269	36.69	
	West	210	25.99	
	Northeast	140	17.26	
Urbanicity	Urban	526	68.67	***
	Rural	223	31.33	

Source: The 2009 National Survey of Federally Qualified Health Centers; HIT-Health Information Technology.

HIT based on health centers’ possession of 16 functionalities of technologies.

* p <.05; ** p <.01; *** p <.001.

### Factors associated with HIT capacity

Several factors had a significant bivariate association with high HIT capacity (Table 3) including incentives for patient satisfaction, overall measure of QI support, having a usual source of care, and regional location. In terms of the quality of care outcomes, usual/often receipt of discharge summaries, patient reminders, and ease of getting timely appointment for specialty care were positively associated with having increased HIT capacity. Other factors that were significantly associated with increased HIT capacity were: having incentives for patient satisfaction (OR=1.47, CI: 1.04, 2.07), having an overall measure of QI support (OR=1.46, CI: 1.10, 1.94), and having a usual source of care (OR=1.75, CI: 1.08, 2.82).

**Table 3:** Factors associated with higher HIT capacity at the FQHCs (n=776)

		**HIT capacity**			
		**OR**	**LCL**	**UCL**	**p-value**
Receipt of discharge summaries	Usual/Often	1.46	1.26	1.70	<0.0001
	Some/Rarely/Never [ref]				
Patient Reminders	Usual/Often	1.65	1.41	1.93	<0.0001
	Some/Rarely/Never [ref]				
Getting timely appointment -specialty care	Easy/somewhat difficult	1.33	1.14	1.56	0.0004
	Very Difficult [ref]				
Incentive for patient satisfaction	Yes	1.47	1.22	1.76	<0.0001
	No [ref]				
HRSA Disparity Coordination	Yes	1.14	0.88	1.48	0.3093
	No				
Overall Measure of QI Support	Yes	1.46	1.26	1.69	<0.0001
	Some or none [ref]				
Physician vacancies	Yes	0.79	0.67	0.95	0.0099
	No [ref]				
Nurse vacancies	Yes	0.76	0.62	0.92	0.0058
	No [ref]				
Hospital Affiliation	All	1.20	1.04	1.39	0.0147
	Some or none [ref]				
Usual Source of Care	Usual/Often	1.75	1.36	2.25	<0.0001
	Some/Rarely/None [ref]				
Percent Medicaid		1.82	1.11	2.97	0.0171
Percent minority	High (50% or more)	1.24	0.99	1.55	0.0624
	Medium (5–49%)	1.42	1.12	1.80	0.0044
	Low (<5%) [ref]				
Size	Large	1.17	0.94	1.46	0.1565
	Medium	0.88	0.75	1.04	0.1258
	Small [ref]				
Region	Midwest	0.51	0.40	0.64	<0.0001
	South	0.78	0.63	0.96	0.0169
	West	0.60	0.49	0.75	<0.0001
	Northeast [ref]				
Urbanicity	Urban	1.28	1.08	1.51	0.0043
	Rural [ref]				

Source: The 2009 National Survey of Federally Qualified Health Centers.

HIT-Health Information Technology.

HIT based on health centers’ possession of 16 functionalities of technologies.

OR-Odds Ratio; CI- Confidence Interval; ref- Reference category.

Odds ratios reported are for having higher odds of increased HIT capacity in relation to the reference category.

p-value is for the association between HIT capacity and outcome measures of interest.

### Unadjusted and adjusted odds for quality of care outcomes

Table 4 reports the unadjusted and adjusted odds ratios for quality of care measures:

**Table 4:** Unadjusted and adjusted odds ratios for associations between health information technology capacity and quality of care in federally qualified health centers for select covariates

		**Receipt of discharge summaries**		**Patient reminders**		**Timely appointment for specialty care**	
		**Unadjusted OR (95% CI)**	**Adjusted OR (95% CI)**	**Unadjusted OR (95% CI)**	**Adjusted OR (95% CI)**	**Unadjusted OR (95% CI)**	**Adjusted OR (95% CI)**
HIT Capacity	High	**1.62**	**1.43**	**1.87**	**1.74**	**1.49**	**1.77**
		(1.34, 1.95)*	(1.01, 2.40)*	(1.55, 2.27)***	(1.23, 2.45)**	(1.23, 1.79)***	(1.24, 2.53)**
	Medium	1.19	1.28	1.04	0.93	**0.74**	1.15
		(0.97, 1.46)	(0.87, 1.88)	(0.83, 1.31)	(0.61, 1.41)	(0.59, 0.94)*	(0.75, 1.75)
	Low [ref]						
Incentive for patient satisfaction	Yes	**1.41**	1.08	**1.86**	**1.49**	1.19	0.97
		(1.17, 1.70)***	(0.74, 1.57)	(1.55, 2.24)***	(1.05, 2.14)*	(0.99, 1.44)	(0.66, 1.42)
	No [ref]						
HRSA Disparity Coordination	Yes	**1.58**	**1.92**	0.91	0.61	0.87	1.11
		(1.22, 2.04)***	(1.21, 3.04)*	(0.69, 1.18)	(0.36, 1.01)	(0.66, 1.13)	(0.65, 1.91)
	No [ref]						
Overall Measure of QI Support	Yes	**1.33**	1.02	**1.45**	1.09	**1.66**	**1.70**
	Some or none [ref]	(1.15, 1.54)***	(0.75, 1.38)	(1.24, 1.69)***	(0.80, 1.50)	(1.42, 1.95)***	(1.23, 2.36)**
Physician vacancies	Yes	1.01	1.21	0.87	0.81	**0.73**	**0.71**
		(0.85, 1.21)	(0.87, 1.68)	(0.72, 1.05)	(0.59, 1.11)	(0.61, 0.88)**	(0.51, 0.99)*
	No [ref]						
Nurse vacancies	Yes	**0.62**	**0.63**	1.01	1.10	**0.78**	0.99
		(0.51, 0.76)***	(0.46, 0.87)**	(0.81, 1.25)	(0.79, 1.52)	(0.63, 0.96)*	(0.71, 1.39)
	No [ref]						
Hospital Affiliation	All	**2.42**	**1.96**	**1.53**	1.17	**1.71**	**1.42**
		(2.08, 2.80)***	(1.44, 2.67)***	(1.31, 1.78)***	(0.85, 1.59)	(1.47, 1.99)***	(1.03, 1.95)*
	Some or none [ref]						
Usual Source of Care	Usual/Often	**1.84**	1.37	**2.34**	1.51	0.99	0.88
		(1.44, 2.34)***	(0.82, 2.67)	(1.73, 3.15)***	(0.87, 2.62)	(0.77, 1.28)	(0.53, 1.46)
	Some/Rarely/None [ref]						
Percent Medicaid		**4.73**	1.41	**3.13**	**3.10**	0.70	**0.09**
		(2.84, 7.86)***	(0.48, 4.12)	(1.88, 5.22)***	(1.02, 9.42)*	(0.42, 1.19)	(0.03, 0.30)***
Percent minority	High (50% or more)	**0.52**	**0.52**	**1.26**	1.20	**0.55**	**0.41**
		(0.41, 0.65)***	(0.31, 0.88)*	(1.01, 1.59)*	(0.74, 1.95)	(0.44, 0.69)***	(0.25, 0.69)**
	Medium (5–49%)	0.84	0.79	1.11	0.73	**0.72**	**0.56**
	Low (<5%) [ref]	(0.66, 1.07)	(0.47, 1.34)	(0.87, 1.42)	(0.45, 1.19)	(0.57, 0.91)**	(0.34, 0.94)*
Size	Large	1.20	1.12	**1.28**	1.04	1.18	**1.92**
		(0.96, 1.50)	(0.71, 1.77)	(1.02, 1.62)*	(0.67, 1.61)	(0.94, 1.49)	(1.22, 3.01)**
	Medium	**1.29**	1.26	**1.27**	1.10	1.07	1.29
		(1.09, 1.51)**	(0.90, 1.77)	(1.07, 1.50)**	(0.79, 1.54)	(0.91, 1.27)	(0.90, 1.84)
	Small [ref]						
Region	Midwest	**0.61**	**0.54**	0.88	1.37	0.98	1.51
		(0.47, 0.79)***	(0.33, 0.90)*	(0.69, 1.13)	(0.85, 2.20)	(0.78, 1.24)	(0.91, 2.50)
	South	**0.36**	**0.28**	0.69	0.71	**0.52**	**0.54**
		(0.29, 0.46)***	(0.17, 0.44)***	(0.56, 0.86)	(0.44, 1.15)	(0.42, 0.64)***	(0.33, 0.89)*
	West	**0.33**	**0.28**	0.89	1.09	**0**.**61**	0.89
		(0.26, 0.41)***	(0.17, 0.44)***	(0.71, 1.12)	(0.68, 1.75)	(**0**.**49**, **0**.**77**)***	(0.55, 1.45)
	Northeast [ref]						
Urbanicity	Urban	0.87	0.87	0.89	**0**.**60**	1.09	**1**.**65**
		(0.74, 1.04)	(0.59, 1.27)	(0.75, 1.06)	(**0**.**42**, **0**.**86**)**	(0.91, 1.30)	(**1**.**11**, **2**.**46**)*
	Rural [ref]						

Source: The 2009 National Survey of Federally Qualified Health Centers; HIT-Health Information Technology.

HIT capacity based on health centers’ possession of 16 functionalities of technologies; QI-Quality Improvement.

Odds ratios compare variables versus their reference category in terms of having improvements in the outcome measures.

OR-Odds Ratio; CI- Confidence Interval; ref-ref correspond to the reference group in the association analysis.

Adjusted odds account for the other variables in the table, which account for: QI initiatives, workforce, usual source of care, as well as demographic variables.

* p <.05; ** p <.01; *** p <.001.

### Receipt of discharge summary

After adjusting for other variables in the model, high HIT capacity facilities had 1.43 times the odds of usually/often receiving discharge summaries (OR=1.43, CI: 1.01, 2.40) and centers with HRSA disparities coordination had a 92% increase in odds of receiving discharge summaries (OR=1.92, CI: 1.21, 3.04). FQHCs with a vacancy of nurses (OR=0.63, CI: 0.46, 0.87) and those with 50% or more minorities (OR=0.52, CI: 0.31, 0.88) had a significant decrease in odds of receiving discharge summaries. Hospital affiliation was associated with nearly a 100% increase in odds of receiving discharge summaries (OR=1.96, CI: 1.44, 2.67). Health Centers in the Midwest (OR=0.54, CI: 0.33, 0.90), Southern (OR=0.29, CI: 0.15, 0.56), and Western (OR=0.32, CI: 0.17, 0.61) regions of the country had decreased odds of receiving discharge summaries compared to the Northeast.

### Patient reminders

FQHCs which had high HIT capacity had nearly 2 times the unadjusted odds of sending patients notifications for preventive or follow-up care (OR=1.87, CI: 1.55, 2.27). The relationship between HIT capacity and the outcome measures remained statistically significant after adjusting for organizational and some individual factors. Having incentive for patient satisfaction increased the odds of having patient reminders by nearly 50% (OR=1.49, CI: 1.05, 2.14). A higher percentage of Medicaid patients was associated with increased odds of having patient reminders (OR=3.10, CI: 1.02, 9.42). Urban centers were less likely to have patient reminders after adjusting for covariates (OR=0.60, CI: 0.42, 0.86).

### Timely appointment for specialty care

FQHCs with high HIT capacity (OR=1.77, CI=1.24, 2.53) and a high overall measure of QI support (OR=1.70, CI: 1.23, 2.36) were more likely to get timely appointments for specialty care. Having physician vacancies decreased the odds of timely appointments for specialty care (OR=0.71, CI: 0.51, 0.99), while centers having hospital affiliations (OR=1.42, CI: 1.03, 1.95) and being a large facility (OR=1.92, CI: 1.03, 3.57) were more likely to have ease in getting timely appointments. For each percentage increase in Medicaid patients, there was a significant reduction in the odds of having timely appointments for specialty care (OR=0.09, CI: 0.03, 0.30). Centers with a higher percentage of minorities were also more likely to have difficulty getting timely appointments for specialty care compared to those with a lower percentage of minorities (OR=0.41, CI: 0.21, 0.84). Regarding geographic location, FQHCs in the South were significantly less likely to have timely appointment for specialty care compared to their regional counterparts (OR=0.54, CI: 0.33, 0.89). Urban facilities on the other hand were more likely to have ease of getting timely appointments for specialty care (OR=1.65, CI: 1.11, 2.46).

## Discussion

Similar to previous studies that have examined the impact of health information technologies and functionalities (i.e., electronic health records, decision-support) on quality of health care, [11,12,14,29-31] we showed that HIT capacity at FQHCs is associated with improved quality of care. Our results showed that high HIT Capacity was significantly associated with increased use of reminders to patients to facilitate follow-up care for preventive services and promote continuous care, receipt of discharge summaries, and timely appointment for specialty care. However, after adjusting for the other control variables, the association between high HIT capacity and the outcome measures dissipated for receipt of discharge summaries and patient reminders. On the other hand, the association was strengthened for timely appointment for specialty care. These findings are promising and suggest that greater adoption and increased capacity of health information technology at FQHCs could help increase the likelihood that patients will realize improved quality of care. This is particularly important because patients who receive reminders from health service providers, including vulnerable patients, have higher rates of cholesterol, breast, and prostate cancer screening, as well as adherence to treatment [32]. In the context of this paper, our results suggest that achieving greater capacity for health information technology is likely to improve quality of services provided by health centers. Additionally, the utilization of HIT and associated computerized processes could increase patient engagement in the care delivery process and continuity of care.

While our findings suggest that HIT has potential benefits for improving quality of care, it also indicates that FQHCs may not be maximizing the potential benefits of health information technology. For example, higher HIT capacity was significantly associated with patient reminders and timely appointments with specialists who operate outside of the health center. However, the magnitude of the association with receipt of discharge summaries was lower. This may be due to lack of collaborations that take full advantage of HIT functionalities, absence of HIT at hospitals where patients are admitted, or HIT systems that cannot be integrated. Addressign each of these possibilities would provide a pathway to increasing meaningful use of HIT and quality of care. We also found significant disparities in FQHCs that serve a larger proportion of minority clients. The relationship between having 50% or more minority clients and declines in quality was observed for receipt of discharge summaries and timely appointment for specialty care. This finding may suggest that participation in the HRSA disparities coordination program improves some indicators of quality of care but not equally for all population subgroups and may not necessarily reduce racial/ethnic disparities. Effective strategies are thus required to ensure that the needs of minority patients are integrated into initiatives to improve quality of care, including the adoption and meaningful use of HIT [15].

Lastly, organizational factors in general have varying influence on different measures of quality. Having all hospital affiliations was significantly associated with improvements in receipt of discharge summaries as well as ease of getting timely appointments for specialty care. However, nurse vacancies, greater proportion of minority clients, and geographic location outside the Northeast U.S. was associated with declines in receipts of discharge summaries [33]. Similarly, physician vacancies, an increasing percent of Medicaid patients, and greater proportion of minority clients was associated with declines in timely appointment for specialty care.

Based on our findings, there are several implications for planning and policy. Firstly, to ensure equity in the benefits of HIT, it is important to assess the capacity of health facilities to provide a series of technology-driven services. It is also important to assess the association of these services to the overall quality of care and care processes within health care organizations. Secondly, HIT should generate information for service providers and patients to improve the care delivery process and quality of care. But, these system should ultimately lead to improvements in patient health outcomes [7]. Thirdly, organizational resources are critical to the effective use of HIT and the effectiveness of coordination programs such as the HRSA disparities initiative on quality of care, especially for the underserved. Establishing hospital affiliations, addressing staff vacancies, and the underserved, i.e., Medicaid and minority patients, appear to be the most important strategy health centers can implement to improve quality of care.

Additionally, the role of organizational characteristics in increasing the benefits of HIT and quality of care has been noted [16,31]. A focus on cultivating relationships with local hospitals, and improving care delivery to all clients may lead to even greater improvements in quality of care. HIT capabilities should be effectively extended to foster collaborations and coordination of care, as well as inform approaches to improving organizational functioning (i.e., tracking and meeting the unique needs of disadvantaged groups). Moreover, understanding and averting the unintended consequences of HIT is essential to realizing the benefits and improving quality of care delivery and patient outcomes [34,35]. Developing strategies and platforms informed by HIT could help facilitate improvements in access to care (i.e., specialty care services) and quality of care [6]. Specifically, Directors of health centers might consider developing more formal arrangements for collaboration and strategies for technical support [36,37].

Lastly, most of the previously published research reveals a positive effect of HIT on care and focused on specific functionalities [38]. As such, there is a need for studies that focus on a broad range of functionalities and provide comprehensive measures of adoption and use of HIT. This will inform requirements for optimal functionalities and use of HIT to improve quality of care, especially for underserved populations. There is also a need for further examination of factors that may facilitate the progressive effect of HIT. As reported by recent studies, [39] a further understanding of how physicians engage with information technology systems, as well as the efficient and effective use of information generated from electronic systems at the point of care and beyond, is essential to optimizing the benefits of HIT.

### Study limitations

The study has several limitations. First, because we did not have access to dates of HIT implementation, we could not determine the number of years that HIT had been in place or the phase of implementation. Previous research shows that phase of HIT, particularly earlier phases of implementation are associated with declines in quality of care, [40] and that the benefits of new technology may take up to fifteen years to be realized [8,11]. Although we could not determine the length of time for which HIT had been in place, the phase of implementation, or establish temporal sequence, the HIT revolution is still in its early stages, thus, the full benefit of HIT is likely to be observed in the future [41]. Additionally, previous studies have suggested that functionalities of HIT and the extent to which HIT is implemented, specifically the capacity of HIT, may be more relevant than the length of time that the HIT system has been in place [42,43]. It may be that FQHCs, with better quality of care, had more favorable operating environments, whether financial or otherwise, and were better positioned to adopt HIT and implement advanced functionalities.

Because our study used a facility survey, we were unable to control for possibly relevant variables such as patient age, socioeconomic status or clinical indicators. Although we used the best available measures and accounted for proportion of minority and Medicaid patients served by the health facilities, these measures may not have been adequate. Additionally, the absence of case mix and other factors by level of HIT capacity could also have resulted in missed small differences between lower and higher capacity adopters of HIT. Given the promise of HIT as a mechanism for improving quality of care and health outcomes, especially for vulnerable populations, the information presented in this study is an important step to identifying areas for further empirical examination and improvement.

## Conclusion

The American Recovery and Reinvestment Act of 2009 (ARRA) investment in health information technology was enacted with the expectation that HIT would improve the care delivery process and quality of care. We examined the relationship between HIT capacity in FQHCs and quality of care and found a positive association between greater HIT capacity and quality of care, measured by receipt of discharge summaries, patient reminder systems for preventive services and follow-up care, and timely appointment for specialty care. Considering the promise of HIT as a tool for improving quality of care, systematic examination of organizational structure and processes that facilitate “meaningful use” must be a key component of health center operations, particularly facilities that serve vulnerable populations [4,15]. These strategies are essential to translating gains from efficiency/intermediate benefits (i.e., improvements in service delivery and quality of care) to effectiveness gains, such as improvements in clinical outcomes [29]. These might include greater utilization of technology that directly influences health outcomes and not just the quality of care.

While technology may facilitate mechanisms that support process improvements, providers must implement systems (i.e., planning, training, technology driven improvements in physician productivity) that materialize technology based benefits [30,35,40]. However, without adequate resources, health centers may not be able to implement technology and provide services that will improve health outcomes. Similarly, there are no acceptable strategies for achieving the benefits of HIT across the U.S. health care system [17]. The effects of HIT on outcomes may be context specific and vary by type of outcome examined [10]. Reviews of the literature have shown that most of the published research showing a positive effect of HIT on care focused on specific components [38]. Our findings present a promising effect of HIT and indicate that future studies must look beyond the capacity of HIT and focus on specific aspects of IT that are more likely to lead to improvements in quality of care and ultimately clinical outcomes. The results also suggest that realizing quality of care benefits from technology may occur over a longer period post HIT implementation. Thus, analysis of the effectiveness of HIT should use time spans longer than that of process measures.

## Abbreviations

HIT: Health Information Technology; FQHC: Federally Qualified Health Center; (HITECH): Health Information Technology for Economic and Clinical Health Act; ARRA: American Recovery and Reinvestment Act; BPHC: Bureau of Primary Health Care.

## Competing interests

The authors declare that they have no competing interests.

## Authors’ contributions

All listed authors participated in multiple aspects of producing the manuscript and made substantial contributions to the manuscript. Authors’ contribution to the manuscript included the following: JAF, SB, and BJ participated in the conception of the study, design, interpretation and writing of the paper. BJ, SB, and JAF led the analysis and interpretation. LMS, PAR, and KP supported the interpretation of the results and editing of the papers. All authors read and approved the final manuscript.

## Pre-publication history

The pre-publication history for this paper can be accessed here:

http://www.biomedcentral.com/1472-6963/13/35/prepub
